# Editorial to 30th anniversary: historical links between the *Psicologia: Reflexão e Crítica* and the postgraduate psychology program at *Universidade Federal do Rio Grande do Sul* (Brazil)

**DOI:** 10.1186/s41155-018-0098-8

**Published:** 2018-07-04

**Authors:** Terezinha Féres-Carneiro, William B. Gomes, Claudio S. Hutz, Eduardo Remor

**Affiliations:** 1 Pontifícia Universidade Católica do Rio de Janeiro, Rio de Janeiro, Brazil; 2 Universidade Federal do Rio Grande do Sul, Porto Alegre, Brazil

We are privileged to write this editorial^1^ at such a significant moment for the Postgraduate Psychology Program at *Universidade Federal do Rio Grande do Sul* in Brazil as we celebrate the 30th anniversary of its first course in 1988. Paired with the beginning of the postgraduate psychology program, our journal was launched, and we would like to take this opportunity to talk about its path as well. In Brazil, many scientific journals have been launched and continue to be associated with higher education institutions. *Psicologia: Reflexão e Crítica* is one of these journals, affiliated with the Postgraduate Program in Psychology of *Universidade Federal do Rio Grande do Sul* (UFRGS). The journal actually appeared in 1986 with a modest regional proposal to discuss current ideas on the relations between psychology as a profession and science, hence the original title in Portuguese of *reflexão* (reflection) and *crítica* (criticism). In 1985, after 31 years of military dictatorship, democracy was reestablished in the country, and universities were discussing the best options for their administrative and academic policies. Thus, *Psicologia: Reflexão e Crítica* emerged, in the midst of these hearty discussions, as a precursor to the Postgraduate Program in Psychology created in 1987 and beginning its activities in March 1988. In its modest proposal, the journal adopted a scientific editorial style and soon attracted articles from researchers from leading Brazilian universities. The growing development of the Postgraduate course in psychology at UFRGS and the recognizable importance of the journal *Psicologia: Reflexão e Crítica* can be related, as the journal played a great role in documenting the history of the program, the diffusion of the research and the discussions regarding the advances in postgraduate studies in psychology in Brazil. Through time, *Psicologia: Reflexão e Crítica* became a singular periodical and a model in our context of pioneering initiatives towards internationalization (Remor, 2016). Mindful of its international vocation, the journal has been published under the SpringerOpen brand by Springer Nature since volume 29 of 2016. The journal also had its name updated in English to reflect better the scope of the journal, to be known as Psicologia: Reflexão e Crítica | Psychology: Research and Review (Remor, 2016).

The first movements and origins of the postgraduate course in psychology may be found in December 1986, when Professor Terezinha Féres-Carneiro was invited to Porto Alegre to give a conference on family therapy at the university. On that occasion, Professors Claudio Hutz and William Gomes had a special meeting with her to elaborate on and plan the creation of the program and to think about the curricular structure based on previous experiences. To readers not familiar with postgraduate study levels in Brazil, professional training occurs in postsecondary education, called undergraduate, or undergrad, which leads to graduate, culminating in the professional degree. Professional licensing comes with the completion of the first 5 years of college, thus advanced postgraduate training in research and teaching grants master’s and doctor’s degrees.

During this meeting, they also talked about the challenges of simultaneously teaching in a postgraduate program, doing research, and being a practitioner (clinical psychologist) and how to reconcile these demands. In addition, they asked about the experience of Professor Féres-Carneiro as coordinator of postgraduate studies in clinical psychology at *Pontifícia Universidade Católica do Rio de Janeiro*, where they had just implemented the doctoral level the previous year (1985). This interview was recorded and published in the first issue of the *Psicologia: Reflexão e Crítica*, entitled “Clinical Practice and Academic Work: An Interview with Psychologist Terezinha Féres-Carneiro” (Gomes & Hutz, 1987).

They were also intrigued with what would be suitable conditions for an institution to organize a postgraduate course in psychology at the master’s and doctoral levels. At that time, the national requirements were that at least 50% of the faculty should teach in the main research concentration area, with the other part teaching in related areas. Today, the requirements are not so rigid, and the emphasis is placed on teaching and research production with the curriculum structured to suit the program’s lines of research.

Recalling the history of postgraduate psychology training in Brazil, we see that, in the 1960s, the only existing master's degree in psychology was at *Pontifícia Universidade Católica do Rio de Janeiro*. In the 1970s, master's degrees were created in the cities of São Paulo, Brasília, Porto Alegre and João Pessoa. In 1978, the evaluation system was instituted by *Coordenação do Aperfeiçoamento do Pessoal do Ensino Superior* (CAPES) in order to better evaluate and monitor the expanding programs, and in 1982, the CAPES agency became consolidated as a government postgraduate studies coordinator. In the 1980s, master’s programs in psychology were implanted in Recife and Natal. In the following decade, Belém, Florianópolis, Belo Horizonte, and Campo Grande added programs. In the 2000s, Salvador, Vitória, Goiânia, and Manaus implemented postgraduate studies. More recently, programs were implemented in Porto Velho and São Luís. Regarding the doctoral level, until 1970, there was no program in the area. In the 1970s, the first doctoral programs were created in São Paulo.

From the 1980s, doctoral programs in psychology emerged in several states, such as the state of *Rio de Janeiro*. In the 1990s, doctoral programs were organized in the states of Rio Grande do Sul, Pará, Rio Grande do Norte, Pernambuco, Espírito Santo, and Brasilia DF. In the 2000s, the states of Santa Catarina, Minas Gerais, Bahia, Goiás, and Ceará created their doctoral programs. Thus, 20 Brazilian states already had postgraduate programs, of which only three did not offer a doctorate degree: Rondônia, Amazonas, and Maranhão.

The goal of implementing psychology postgraduate programs in all Brazilian states has been longstanding (Féres-Carneiro, Bastos, Feitosa, Seidl-de-Moura, & Yamamoto, 2010). Today, this goal has almost been reached. Only six of the 26 states of the federation do not have postgraduate studies in psychology: Roraima, Amapá, Acre (Northern region), Mato Grosso and Tocantins (Center-west region), and Piauí (Northeast region). In total, we now have 84 postgraduate programs in psychology in Brazil. Forty are in the Southeast, 17 in the Northeast, 11 in the South, 11 in the Center-west, and 5 in the North. Of the 84 programs, 53 have master’s and doctorate levels, and 31 of them only have the master’s level, with 25 academic master’s and six professional master’s.

In addition, as noted by Féres-Carneiro et al. (2010), the creation of professional master’s programs coupled with already consolidated academic postgraduate programs was one of the goals of academics. A professional master’s degree is an applied modality of postgraduate studies, whose objective is to contribute to the national productive sector, offering advanced training in techniques, processes, or themes that meets the labor market demand. For a long time, the area was refractory to considering the possibility of proposing a professional master’s program. Thus, the first program was created in 2013. Currently, there are nine approved professional master’s programs, although only three have been included in the last 4-year evaluation period. These programs are evaluated using criteria different from those used to evaluate academic programs. The states with programs in operation or in preparation include *Rio de Janeiro* (three), *São Paulo* (three), *Pernambuco* (two), and *Rio Grande do Norte* (one).

Returning to the history of the Postgraduate Program in Psychology of *Universidade Federal do Rio Grande do Sul*, which is one of the oldest program, the course has emerged as a center of excellence in research and the training of teacher-researchers, with extensive international placement, since being established. In 1988, the program had only five faculty members. Six years after the implementation of the master’s program, in 1994, the doctorate program was started and had 12 permanent faculty members and two collaborators.

The program’s continued growth was recognized by the excellent evaluations obtained from the CAPES agency. In the triennium 1998–2000, the Postgraduate Program in Psychology of UFRGS earned a score of “five;” in the following period, it earned a score of “six;” and since the triennium 2004–2006, it has maintained a score of “seven,” the maximum score of the CAPES evaluation.

A couple of years after the program’s twentieth anniversary, professors William Gomes and Claudio Hutz published an article entitled “Historical and conceptual notes on the Graduate Program in Psychology of the *Universidade Federal do Rio Grande do Sul*” in *Psicologia: Reflexão e Crítica*; the article documented information of value about the program’s growth. At that point, the course had 18 faculties, 17 with the distinction of having received a *Conselho Nacional de Desenvolvimento Científico e Tecnológico* (CNPq) researcher productivity scholarship (Gomes & Hutz, 2010). Surprisingly, the largest set of CNPq productivity researchers in a single program was in the Postgraduate Program in Psychology of UFRGS. Today, the program has 20 permanent and three collaborating faculty members. Among the permanent professors, 17 are CNPq productivity scholarship holders, continuing the tradition of having the largest contingent of scholars in a single program.

When created 30 years ago as a master’s degree in developmental psychology, the program had a single line of research and a curricular structure composed of six compulsory courses and 15 electives, of which the student should study five. Today, counting the master’s and doctoral degrees in psychology, after extensive internal discussion held in 2015, the program is organized into four lines of research: (1) psychological assessment and measurement; (2) human development; (3) health, prevention, and intervention; and (4) cognitive and behavioral neuroscience. The journal *Psicologia: Reflexão e Crítica* has four sections with a specific scope to be analogous to the fields of expertise of our postgraduate program.

To expand the lines of research, the curriculum is now organized around four major axes: thematic topics, methodology, academic, and complementary skills. The disciplines are offered in three modalities—obligatory, alternative, and elective—, a structure that bears a resemblance to the course’s initial proposal, despite the difference in relation to the multiple and innovative current developments.

Thus, the Postgraduate Program in Psychology of *Universidade Federal do Rio Grande do Sul* followed the growth of the country. In 2012, the number of programs evaluated by CAPES was 69; this number jumped to 84 in 2016. As a result, there was also an increase in the number of faculty members, which went from 1212 to 1558 in the same period. The postgraduate programs in the area have from 9 to 43 faculty members, with an average of 18.5 faculty members per program. Permanent faculty members represent 77% of the professors in Brazilian programs, with the remaining being visiting or collaborating professors. The intellectual production of the area has also greatly increased, increasing by 80% in the last 4 years, compared to the previous triennia, the period of years previously used in the CAPES evaluation (currently, evaluations are carried out every 4 years). Currently, the Postgraduate Program in Psychology of UFRGS has 23 faculties, which is higher than the faculty average per program in Brazil. The stability of the faculty staff is worth noting. Three of the founders, professors Claudio Hutz, William Gomes and Cesar Piccinini remain in the program to this day. Others hired in the 1990s and 2000s remain in the program. The program has also sought to renew its faculty by incorporating young, productive researchers.

In the last CAPES assessment, the overall H-Score (Google Scholar) for the programs in the area was 9.26, and programs with H-scores above 10.5 are considered “Very Good” or as having a faculty with high maturity (Bastos, Tomanari, & Trindade, 2017). The faculty of the Postgraduate Program in Psychology of *Universidade Federal do Rio Grande do Sul* has the highest average H-Score among all the programs in the area: 25.8 (almost three times higher than the average of among the programs). It was also assessed to have a remarkable academic maturity and high qualification, a quality common to its professors. In addition, several professors hold important positions in the decision-making committees of development agencies such as CAPES and CNPq, in the directorates of scientific societies, with publishers of national and international journals, and in the coordination research groups at the *Associação Nacional de Pesquisa e Pós-graduação em Psicologia* (ANPEPP). In addition, the majority of its professors are involved in some form of international exchange linked to their research projects.

Moreover, according to the 2017 Quadrennial Report (Bastos et al., 2017), the intellectual production, both by quantity and quality, of the Postgraduate Program in Psychology of *Universidade Federal do Rio Grande do Sul* is the highest in the area. The program also held this status in previous triennia and the most recent 4-year evaluation, indicating the excellence level of the program. The average contribution per permanent faculty per year was 397 points, near the maximum possible value of 400, based on the table of best production per year (in this classification, only the highest evaluated journals count). The average quality of articles, books, and chapters was 96, which is equivalent to the top classification for high-quality chapters and books. This production is fairly well distributed among the faculty, and 85% of the faculty members produced above average for programs at the top of the CAPES assessment scale. In addition, production is highly internationalized. Approximately 84% of the articles were published in international journals or in English, in higher nationally evaluated journals (*Qualis* first and second quartiles). Thus, the last CAPES evaluation noted that the Postgraduate Program in Psychology of *Universidade Federal do Rio Grande do Sul* had the most outstanding scientific production in psychology in Brazil. With all these highlights, even in relation to other programs awarded a score of 7 by CAPES (that is, only two others), we can say that the Postgraduate Program in Psychology of UFRGS is one of the best programs in the area, standing out from so many others, including some traditional programs, getting the highest score in the evaluations since 2004.

Another historical dimension of postgraduate studies in psychology in the country where the Postgraduate Program in Psychology of UFRGS played a very important role was the collaboration with ANPEPP. In 1988, the first symposium of the ANPEPP took place in Caruaru, PE, organized by Ana Lucia Schliemann, who was at that time a professor at the *Universidade Federal de Pernambuco*. Twelve programs participated in the symposium, and each program could send up to three delegates. For *Pontifícia Universidade Católica do Rio de Janeiro*, the delegates were Circe Navarro Vital Brasil (1930–1995), Ana Maria Nicolaci, and Teresinha Féres-Carneiro. For *Universidade Federal do Rio Grande do Sul*, they were Ângela Maria Brasil Biaggio (1940–2003), William Gomes, and Claudio Hutz. At the time, the symposia were scheduled to take place every year. In 1989, professors Claudio Hutz and William Gomes, organized the second symposium in Gramado, RS, proposing an innovative format that is maintained to this day and is implemented for other scientific events in the country. The format of working groups (“*grupos de trabalho*”, i.e., network groups of researchers and faculty from different regions of the country with research and interest in common topics) has been very successful and continues to this day. At the second symposium, there were 10 working groups, who gathered with great eagerness in Gramado. In 1990, the third symposium took place in Águas de São Pedro, SP, and from then on, they were held every two years (even-numbered years). The number of ANPEPP working groups can easily evidence the growth of the area over the last 30 years. The second symposium held in Gramado, RS, in 1989 had 10 registered working groups. For the seventeenth symposium (2018), in Brasilia, DF, 81 working groups registered, and 79 have currently been approved.

The journal *Psicologia: Reflexão e Crítica* has represented all these changes and advances as a kind of public notary, documenting the facts as they occur. As an example, the journal published a special issue in 2015 that included 13 articles and documented the dialogs with researchers from other countries from the ANPEPP’s Forum around issues facing Brazilian scientific policies, including analysis of international relations on research and publication by scholars from different countries, evaluation of English language in articles of the top five Brazilian psychology journals, a detailed description of the Brazilian postgraduate education system for the non-Brazilian world, and a description of ANPEPP’s Symposium from the perspective of an observer from the American Psychological Association (Gomes & Fradkin, 2015).

In recent years, with the full professionalization of the journal *Psicologia: Reflexão e Crítica*, the diffusion and metrics of the journal (i.e., impact factor, altmetrics, usage factor, and social media impact) have been consistently increasing. Along with an increasing interest from international researchers to publish in the journal. Recent data provided by Google analytics (June 2018) indicate a 35% increase in visits to our main website (https://prc.springeropen.com/) from 2016 to 2017. The ten countries with the highest access to the website in 2017 were (data within parenthesis indicates proportion in comparison with other countries) Brazil (26.7%), the United States (15.9%), the United Kingdom (7.5%), India (5.4%), Portugal (4.9%), Spain (2.6%), Australia (3.2%), Canada (2.2%), Philippines (1.9%), and the Netherlands (2.0%).

The tireless work of the current and former editors of *Psicologia: Reflexão e Crítica* over its 31 years of existence is an important piece of its success. This journal has been one of the most important publications on psychology in the country, presenting Brazilian research in psychology to the world abroad and being one of the few Brazilian journals ranking in the first quartile (“A1”) in the area’s *Qualis* (CAPES journal ranking).

Finally, we would like to pay tribute to the founders of the Postgraduate Program in Psychology of *Universidade Federal do Rio Grande do Sul*, Professors Claudio Hutz, William Gomes, Cesar Piccinini, and the retired professors Tânia Mara Sperb and Maria Alice de Mattos Parente, who also contributed so much to the research lines on human development and neuropsychology, respectively. In particular, we would like to mention the dear teacher to many among us, Ângela Maria Brasil Biaggio. Professor Biaggio was a pioneer in developmental psychology research in the country. Unfortunately, she is no longer with us, but she gave us, like so many other great masters, an inheritance. She, like the great masters, taught us that this heritage can be transformed, leading us to be unafraid to dare and innovate, a legacy that we too must leave to our students and to the new generations, in the exercise of our gratifying profession to prepare them well for teaching and for research.

## Additional file

Additional file 1: 30th Anniversary Ceremony of the Postgraduate Course in Psychology at the UFRGS (Brazil), March 12, 2018. (DOCX 35 kb)

## Footnotes

1 This editorial was inspired by the opening conference of Terezinha Féres-Carneiro at the 30th Anniversary Ceremony of the Postgraduate Course in Psychology at the UFRGS (Brazil), March 12, 2018. The complete conference transcript in the original Portuguese is included as Additional file 1.
