# Supplementary material for: Editorial to 30th anniversary: historical links between the Psicologia: Reflexão e Crítica and the postgraduate psychology program at Universidade Federal do Rio Grande do Sul (Brazil)
Source: Psicol Reflex Crit. 2018 Jul 4;31:18. doi: 10.1186/s41155-018-0098-8 (PMC6966914; doi:10.1186/s41155-018-0098-8)
Supplement: Supplementary file 1 — 30th Anniversary Ceremony of the Postgraduate Course in Psychology at the UFRGS (Brazil), March 12, 2018. (DOCX 35 kb) [file 41155_2018_98_MOESM1_ESM.docx]

Conferência de Abertura, 12 de Março de 2018. Cerimônia do 30º Aniversário do *Programa de Pós-graduação em Psicologia* da *Universidade Federal do Rio Grande do Sul*. Porto Alegre, RS, Brasil.

**30 anos de história do Programa de Pós-graduação em Psicologia da UFRGS no contexto do desenvolvimento da Pós-Graduação em Psicologia no Brasil**

Terezinha Féres-Carneiro^[[1]](#footnote-1)^

*Pontifícia Universidade Católica do Rio de Janeiro*, Brasil

Foi com imensa alegria que aceitei o convite do Programa de Pós-graduação em Psicologia da UFRGS para proferir esta Conferência de Abertura, na comemoração dos seus 30 anos. Quero agradecer a cada um dos professores do Programa, por esta honra, e de modo especial ao professor Claudio Hutz, que me transmitiu com carinho o convite do Programa, ao professor William, que me enviou rico material para reavivar minha memória; e ao professor Marco Teixeira, coordenador do Programa, pelo envio do Relatório CAPES, referente ao último quadriênio, solicitado por mim.

Os vínculos que me unem ao PPG em Psicologia da UFRGS datam da época em que ele ainda estava sendo gestado. Nossa história teve início em dezembro de 1986, quando estive em Porto Alegre para proferir uma conferência sobre Terapia de Família na 2ª Programação Cultural da UFRGS. Lá se vão mais de 31 anos e ainda tenho viva, na memória, a vivência do dia em que aqui estive, convidada pelos professores Cláudio e William, para avaliar o projeto de implantação da Pós-graduação *stricto sensu*. Além desta consultoria sobre o projeto do curso, os professores queriam também conversar comigo sobre minha experiência como psicóloga clínica e pesquisadora.

Fazia muito calor em Porto Alegre. William vestia uma bermuda e carregava um gravador do tamanho de uma impressora multifuncional, daqueles que só quem viveu naquela época conheceu, e caminhávamos pelo campus em busca de uma sala onde iríamos trabalhar. O clima era de muito entusiasmo e os dois jovens doutores queriam me perguntar muitas coisas, mas esperavam, sobretudo, que eu avaliasse o projeto de implantação do Mestrado, com atenção especial para a sua estrutura curricular, e que desvendasse para eles, o que era, à época, pelo menos no Sul do país, quase um mistério: como conciliar a prática clínica com a atividade acadêmica?

Da primeira parte da nossa conversa, referente ao projeto de implantação do curso de mestrado, lembro-me com clareza, que ao discutir com os dois jovens doutores a estrutura curricular do curso a ser implantado, enfatizei a necessidade de as disciplinas instrumentais, relacionadas à metodologia científica e aos métodos de pesquisa serem disciplinas obrigatórias. A estrutura curricular da primeira proposta do curso, tinha como disciplinas obrigatórias: Epistemologia e Metodologia Científica, Seminário de Pesquisa, Métodos Quantitativos ou Métodos Qualitativos, além de Teorias em Desenvolvimento Psicológico e Seminário Avançado em Desenvolvimento Psicológico. Dentre as 15 disciplinas eletivas (quase todas com temáticas relacionadas ao Desenvolvimento Humano), o aluno deveria cursar cinco. Na sua organização inicial, o programa contava com uma única linha de pesquisa, Desenvolvimento Social e Cognitivo, possibilitando grande liberdade conceitual e metodológica, liberdade esta que se mantém até os dias de hoje no PPG.

A segunda parte da conversa constituiu-se numa entrevista sobre minha prática clínica e meu trabalho na academia. Nesta entrevista, foram abordados dois temas principais. O primeiro deles dizia respeito a algo que o professor William considerava atípico, pelo menos na realidade sulista, ou seja, meu grande envolvimento com a universidade, com a pós-graduação e a pesquisa, e, ao mesmo tempo, a manutenção das minhas atividades como psicóloga clínica. O segundo tema, levantado pelos jovens Claudio e William, se referia às condições necessárias para que uma instituição organizasse um Programa de Psicologia Clínica em nível de mestrado e de doutorado. Naquele momento eu era coordenadora do curso de pós-graduação em Psicologia Clínica da PUC-Rio, onde tínhamos acabado de implantar no ano anterior (1985) o nível de Doutorado. Essa entrevista foi gravada e publicada no primeiro número da recém-criada revista *Psicologia: Reflexão e Crítica* (Gomes & Hutz, 1987), com o título "*A prática clínica e o trabalho acadêmico: Uma entrevista com a psicóloga Terezinha Féres Carneiro*".

Acho que vale a pena relatar, resumidamente, algumas respostas a essas questões, tal como entendidas há mais de 30 anos atrás, para que, aceitando hoje a sugestão do professor William, eu possa narrar nesta Conferência, um pouco a minha percepção de como a pós-graduação em Psicologia foi se configurando e evoluindo ao longo do tempo no país, e de como o PPG em Psicologia da UFRGS surgiu e se desenvolveu neste contexto.

Em relação à primeira parte da entrevista, relacionada à questão do envolvimento acadêmico e da atividade clínica, minha resposta à época foi a mesma que eu daria hoje. A diferença hoje, talvez, estivesse na pergunta, na medida em que, me parece que isto talvez não seja mais tão atípico quanto parecia naquele momento. Para mim, a dicotomia entre atividade de pesquisa e atividade clínica é uma falsa dicotomia. Estas duas atividades não são mutuamente excludentes, pelo contrário elas se retroalimentam. Como ensinar e produzir conhecimento em psicologia clínica sem ser psicólogo clínico? Sem exercer a atividade clínica? Desde o meu curso de graduação na PUC-Rio - que sempre teve uma forte tradição em Psicologia Clínica - iniciado em 1968 (portanto há 50 anos atrás), a articulação destas duas atividades sempre me pareceu natural. Tive excelentes professores que exerciam a clínica e eram pesquisadores. Talvez o fato de a PUC-Rio nunca ter tido entre as suas possibilidades de regime de trabalho a “dedicação exclusiva”, mas, apenas, o “tempo integral” (que também não significa estar o tempo todo presente na universidade, mas cumprir com dedicação e competência todas as tarefas esperadas do corpo docente), tenha permitido aos professores-pesquisadores maior liberdade para o exercício profissional dentro de determinados limites, o que contribuiu, e continua contribuindo, para alimentar as atividades de ensino e de pesquisa em psicologia clínica.

Quanto à segunda parte da conversa, relacionada às condições para que uma instituição organizasse um programa de Pós-graduação em Psicologia Clínica, em nível de mestrado e de doutorado, à época, era necessário que o corpo docente tivesse pelo menos 50% dos professores na área de concentração do programa, ou seja, com doutorado em psicologia clínica, e os demais, em áreas afins. Hoje isto não é tão rígido assim, e a ênfase é dada à articulação da produção docente e discente, e da estrutura curricular às linhas de pesquisa do Programa. Como, à época, não havia muitos doutores em Psicologia Clínica no país, era possível também que professores–pesquisadores sem o título de doutor, mas com muita experiência e com produção científica, fossem reconhecidos pelo Conselho Federal de Educação como “Notório Saber”, que tinha uma equivalência ao título de doutor. Um dos fundadores do nosso programa, o psiquiatra e psicanalista Carlos Paes de Barros, teve esta equivalência concedida pelo MEC. Com a grande expansão dos doutorados na área, inclusive, dos doutorados em Psicologia Clínica, hoje, isto não se faz mais necessário.

O Curso de Mestrado em Psicologia Clínica da PUC-Rio foi o primeiro a ser implantado no país, na área da Psicologia, em 1966. Todavia, no nosso PPG, o nível de doutorado só foi iniciado quase 20 anos depois, em 1985, quando eu exercia o cargo de Coordenadora. Foram algumas idas a Brasília, no ano de 1984, para discutir na CAPES, com Rosana Arcoverde, então responsável pela área de Avaliação, o projeto de implantação do Doutorado, que foi aprovado nesse ano, e iniciado em março do ano seguinte. O atraso na implantação do nível de doutorado se deveu a muitos altos e baixos ocasionados por crises internas no programa. Altos que levaram o Programa pioneiro a ser considerado, por muito tempo, como centro de excelência (com a avaliação mais alta concedida pela CAPES); e baixos que o fizeram, por duas vezes, perder o credenciamento. Coordenei o Programa por três vezes, totalizando 14 anos. Em duas destas vezes os colegas me indicaram para a coordenação com a missão de recuperar avaliação do PPG. Foram necessárias muitas reformulações e muitas experiências inovadoras, além de muitos procedimentos pioneiros para recuperar o programa e resgatar o lugar de liderança que, por tanto tempo coube a ele no cenário da Pós-graduação em Psicologia no país.

Retomando um pouco da evolução da pós-graduação em Psicologia no país, vemos que, na década de 1960, o mestrado em Psicologia da PUC-Rio era o único existente no Brasil. Na década de 1970 foram criados cursos de mestrados, em São Paulo, Distrito Federal, Rio Grande do Sul e Paraíba. Em 1978 foi instituída a sistemática de avaliação pela CAPES, para melhor acompanhamento dos cursos em expansão, tendo a agência se consolidado como gestora da pós-graduação em 1982.

Nos anos 1980, mestrados em psicologia foram implantados em Pernambuco e no Rio Grande do Norte. Na década seguinte, de 1990, foi a vez de Pará, Santa Catarina, Minas Gerais e Mato Grosso do Sul. Nos anos 2000, Bahia, Espírito Santo e Goiás e Amazonas implantaram a pós-graduação. E mais recentemente, Roraima e Maranhão.

Em relação ao nível de Doutorado, até 1970, não havia nenhum curso na área. Na década de 1970, foram criados, então, os primeiros cursos de doutorado em São Paulo.

Em 1979, participei, como candidata, das seleções ao doutorado na USP e na PUC/SP. Na USP, onde só havia o nível de doutorado nas áreas de Psicologia Escolar e Psicologia Social, fui entrevistada pelo professor Arrigo Angelini. Na PUC-SP, que oferecia o nível de doutorado nas áreas de Psicologia Clínica, Psicologia Social e Psicologia Educacional, fui entrevistada pela professora Aniella Grinsberg. Aprovada nas duas seleções, escolhi o Doutorado de Psicologia Clínica da PUC/SP, área na qual eu atuava e já havia feito o Mestrado. Titulei-me em agosto de 1981 (em 29 meses), e até hoje, os colegas da PUC/SP me dizem que foi o doutorado mais rápido já realizado no Programa. Sobretudo numa época em que os prazos de realização do doutorado eram muito longos (chegavam às vezes a oito anos). Hoje, o tempo médio da área para realização do mestrado é de 26,6 meses, mas 30 meses é o tempo máximo que a CAPES considera “muito bom”. Para o doutorado, o tempo médio da área é de 48,8 meses, mas até 50 meses a CAPES considera um tempo “muito bom”.

Nos anos 1980, foram implantados cursos de doutorado no Rio de Janeiro. E nos de 1990, no Rio Grande do Sul, Distrito Federal, Pará, Rio Grande do Norte, Pernambuco e Espírito Santo. Na década de 2000, em Santa Catarina, Minas Gerais, Bahia, Goiás e Ceará. Dentre os 20 estados da federação e o DF que têm pós-graduação, só três não têm o nível de doutorado: Roraima, Amazonas e Maranhão.

Quando em 2010, publicamos, Virgílio Bastos, Maria Ângela Feitosa, Maria Lucia Seildl, Oswaldo Yamamoto e eu, na revista *Psicologia Reflexão e Crítica,* o artigo intitulado “Lacunas, metas e condições para a expansão da pós-graduação em Psicologia no país”, propusemos, como uma das metas para a pós-graduação no Brasil, a implantação de curso de pós-graduação em Psicologia em todos os estados da federação.

Hoje esta meta está quase atingida. Apenas seis estados da federação, todos menos um, das regiões Norte e Centro-Oeste, não possuem cursos de pós-graduação em psicologia: Rio Branco, Amapá, Acre, Mato Grosso, Tocantins e Piauí (o único estado da região Nordeste que não possui pós-graduação na área).

No total, temos hoje 84 Programas de pós-graduação em Psicologia no Brasil, assim distribuídos: 40 na região Sudeste, 17 no Nordeste, 11 no Sul, 11 no Centro-Oeste e 5 na região Norte. Dos 84 Programas, 53 têm nível de Mestrado e Doutorado, e 31 deles só têm o nível de Mestrado, sendo 25 Mestrados Acadêmicos e 6 Mestrados Profissionais avaliados.

Dentre as metas colocadas no nosso artigo de 2010, estava a criação de mestrados profissionais, acoplados a Programas já consolidados. Por muito tempo, a área foi refratária à ideia de considerar a possibilidade de propor mestrados profissionais. Assim, eles são muito recentes na área, tendo sido criados a partir de 2013. Atualmente existem nove aprovados (embora só três tenham sido avaliados) e em funcionamento: três no Rio de Janeiro, três em São Paulo, dois em Pernambuco e um no Rio Grande do Norte, que são avaliados com parâmetros distintos dos de programas acadêmicos, dadas as suas especificidades e o fato de ainda não estarem consolidados.

Voltando à história do curso, que agora entra para o rol dos balzaquianos, diferentemente do PPG em Psicologia Clínica da PUC-Rio, que só iniciou o Doutorado quase 20 anos depois da implantação do Mestrado (tendo em vista todas as oscilações que sofreu, podendo ser considerado um programa resiliente), o PPG em Psicologia da UFRGS, desde a sua implantação, já despontou como um centro de excelência na pesquisa e na formação de docentes-pesquisadores, e com expressiva inserção internacional. Em 1988, quando foi iniciado, o Programa contava no seu quadro permanente com apenas cinco professores. Seis anos após a implantação do mestrado, portanto em 1994, o PPG já iniciou o doutorado, e já contava com 12 professores permanentes e dois colaboradores.

O contínuo crescimento do programa resultou nas excelentes avaliações que vem recebendo da CAPES. No triênio 1998-2000 o curso foi avaliado com a nota 5, no triênio seguinte, com a nota 6, e desde o triênio 2004-2006, quando obteve a nota 7, o programa vem mantendo a nota máxima da avaliação da CAPES. Este sucesso vem sendo acompanhado de perto por mim, uma espécie de madrinha do programa, com muito orgulho e muita alegria. Em março de 2008, há exatamente 10 anos, quando o PPG em Psicologia da UFRGS completava 20 anos, fui também convidada para proferir a Conferência Inaugural, que teve como tema: “Pesquisa e prática clínica: construindo articulações teóricas”, ocasião em que contei também um pouco da história que me une ao programa. Esta conferência está publicada na revista *Psicologia Reflexão e Crítica* (Féres-Carneiro, 2008).

Em 2010, quando os professores William e Claudio publicaram, também na revista *Psicologia Reflexão e Crítica,* o artigo intitulado “Anotações históricas e conceituais sobre o Programa de Pós-graduação em Psicologia da Universidade Federal do Rio Grande do Sul”, o programa já havia ampliado seu corpo docente para18 professores, 17 dos quais bolsistas de produtividade do CNPq, o que é considerado, na área, como o maior conjunto de bolsistas de produtividade no mesmo programa.

Hoje o PPG em Psicologia da UFRGS conta com 20 docentes no seu quadro permanente, e com três professores colaboradores. Dentre os docentes permanentes, 17 são bolsistas de produtividade, mantendo a louvável característica de continuar tendo o maior contingente de bolsistas no mesmo programa.

Nascido há 30 anos atrás como Mestrado em Psicologia do Desenvolvimento, o programa tinha à época uma única linha de pesquisa e uma estrutura curricular composta de seis disciplinas obrigatórias e 15 eletivas das quais o aluno deveria cursar cinco. Hoje, ele é organizado a partir de quatro linhas de pesquisa: (1) avaliação e medidas em Psicologia; (2) desenvolvimento humano; (3) saúde, prevenção e intervenção; e (4) neurociência cognitiva e comportamental, reformuladas em 2015, depois de amplo processo de discussão interna.

Tendo em vista a ampliação das linhas de pesquisa, o currículo está organizado em torno de grandes quatro eixos: temático; metodológico; acadêmico; e de competências complementares. E as disciplinas são ofertadas em três modalidades: obrigatórias, obrigatório-alternativas e eletivas; o que guarda uma semelhança com a proposta inicial do curso (que tinha Métodos Quantitativos e Métodos Qualitativos como obrigatórias alternativas), apesar da diferença em relação aos múltiplos e inovadores desdobramentos atuais.

Este crescimento, em quantidade e qualidade, do PPG da UFRGS acompanhou o crescimento da área no país. Em 2012, o número de Programas avaliados na área era de 69. Este número pulou para 84 Programas, em 2016. Consequentemente, houve também um crescimento no número de docentes que passou, no mesmo período, de 1.212 para 1.558. No âmbito da Pós-graduação na área, os Programas variam de nove a 43 docentes, com uma média de 18,5 professores por Programa. Os docentes permanentes representam 77% dos professores nos Programas. A produção intelectual da área também vem crescendo muito, tendo havido um aumento da ordem de 80% no último quadriênio, em relação ao triênio anterior.

Com 23 professores, o PPG da UFRGS está acima do número médio da área de docentes por Programa. Chama atenção a estabilidade do corpo docente. Três dos fundadores - professores Claudio Hutz, William Gomes e Cesar Picinini- e a professora Tania Sperb, contratada um ano depois do início do curso, permanecem até hoje no Programa. Assim como os professores contratados nas décadas de 1990 e de 2000. Por outro lado, o programa tem procurado renovar seu corpo docente incorporando jovens pesquisadores produtivos.

Na última avaliação da CAPES, a média geral do Escore H (Google Scholar) dos Programas da área ficou em 9,26, e Programas com Escore H superior a 10,5 foram considerados como “Muito Bom” (MB) ou de elevada maturidade do seu corpo docente. O corpo docente do PPG da UFRGS é o que possui o Escore H médio mais elevado entre todos os programas da área, ou seja, 25.8 (quase três vezes maior que a média dos Programas). O PPG foi considerado também como tendo notável maturidade acadêmica e com alta qualificação, e nível de qualidade bastante homogêneo entre os docentes.

Além disto, vários docentes do Programa ocupam postos importantes nas comissões decisórias de agências de fomento, nas diretorias de sociedades científicas, na editoria de periódicos nacionais e internacionais, e na coordenação de grupos de pesquisa da ANPEPP, estando a maioria dos docentes envolvida em alguma modalidade de intercâmbio internacional vinculada aos seus projetos de pesquisa.

Em relação à produção intelectual, tanto nos indicadores de quantidade, quanto nos de qualidade, o PPG em Psicologia da UFRGS possui o desempenho mais alto da área. Este cenário de destaque ocorreu em triênios anteriores e se manteve no último quadriênio, indicando o nível de excelência do Programa. A contribuição média por docente permanente por ano foi de 397 pontos, praticamente o valor máximo possível de 400, considerando a tabela de melhor produção por ano. A qualidade média dos artigos, livros/capítulos foi 96, o que equivale à classificação de revistas A1/A2 e de livros L4/L3. Esta produção é bastante bem distribuída entre os docentes. Um contingente de 85% dos docentes produziu acima da média dos programas situados no topo da escala de avaliação. Além disto, a produção é altamente internacionalizada. Aproximadamente 84% da produção foi veiculada em periódicos internacionais ou em inglês, em periódicos nacionais A1/A2. Assim, a última avaliação da CAPES ressaltou que, no quesito Produção Intelectual, o desempenho do PPG em Psicologia da UFRGS foi o mais destacado da área. Com todos esses destaques, mesmo em relação a outros programas avaliados como 7 pela CAPES (são só mais dois), podemos afirmar que o PPG da UFRGS é o melhor programa da área. Felicito com alegria professores, alunos e funcionários, que com competência, dedicação e esforço conjunto contribuem para que o PPG tenha todo este sucesso. Parabéns a todos!

Gostaria de abordar ainda uma outra dimensão da história da pós-graduação no país, em que o PPG da UFRGS teve um importantíssimo papel. Em 1988, ocorreu o I Simpósio da ANPEPP, em Caruaru, PE, organizado por Ana Lucia Schlieming, do qual participaram os doze programas de pós-graduação existentes à época, que podiam ser representados, cada um, por até três professores. Éramos em torno de 25 participantes. Pela PUC-Rio estávamos Circe Navarro Vital Brasil, Ana Maria Nicolaci e eu; e pela UFRGS estavam Ângela Biaggio, e os jovens doutores William Gomes e Claudio Hutz. À época os Simpósios estavam programados para acontecerem a cada ano. Em 1989, os entusiastas professores, Claudio e William, que haviam acabado de implantar o mestrado na UFRGS, organizaram, em Gramado, o II Simpósio com um formato inovador, que é mantido até os dias de hoje. Esse Simpósio foi proposto pelos jovens doutores no formato de Grupos de Trabalho (os GTs da ANPEPP), que tiveram grande sucesso, e se mantêm até hoje. No II Simpósio havia 10 GTs, reunidos com grande entusiasmo em Gramado. Em 1990, o III Simpósio ocorreu em Águas de São Pedro, SP, e a partir daí, passaram a ocorrer de dois em dois anos (nos anos pares).

O grande crescimento da área, nos últimos 30 anos, fica bastante evidente, também se compararmos os dados do II Simpósio, ocorrido em Gramado em 1989, no qual havia 10 GTs, com aqueles que temos em relação ao XVII Simpósio, a ser realizado, em Brasília, de 15 a 18 de julho deste ano, no qual foram inscritos 81 GTs, tendo sido aprovados, até o momento, 79.

Vou me permitir, neste momento, repetir algo que já falei quando aqui estive, em 2008, proferindo a Conferência de Abertura, na comemoração dos 20 anos do Programa. Trata-se de um sábio provérbio chinês ao qual fiz referência à época, mas que tem para mim um significado ainda maior, 10 anos depois, quando encontro o Programa num nível de excelência que eu nem imaginava poder ser alcançado. Diz o provérbio que "a maior alegria do mestre é ver o discípulo ultrapassá-lo". Se ocupei um pouco o lugar de mestre, no final do ano de 1986, e pude agregar alguma coisa da minha experiência em Pós-graduação ao Projeto do Programa que estava por nascer; ver, hoje, este Programa ultrapassar tantos e tantos outros, alguns dos quais, tradicionais, como aquele do qual faço parte, e estar sendo avaliado desde o triênio 2004-2006 (portanto há 14 anos), com a nota máxima do sistema de avaliação da CAPES, é, para mim, fonte de indescritível alegria; da genuína e verdadeira alegria que inunda o mestre ao ver o discípulo ultrapassá-lo.

Sinto-me orgulhosa de ter participado um pouquinho da história deste Programa que é o melhor PPG da área de Psicologia do país. Parabenizo todos aqueles, professores, alunos e funcionários, que colaboraram na construção deste lindo e exemplar percurso, pelo cuidadoso trabalho realizado e pelo grande sucesso alcançado!

Gostaria de parabenizar, também, os atuais e os ex-editores da Revista *Psicologia: Reflexão e Crítica,* pelo incansável trabalho realizado nestes 31 anos de existência, ao longo dos quais tem sido um dos mais importantes veículos de publicação da produção em psicologia no país e no exterior, sendo uma das poucas revistas avaliadas como A1, no *Qualis*da área.

Para finalizar, gostaria de prestar uma homenagem aos fundadores do PPG em Psicologia da UFRGS, professores Claudio Hutz, William Gomes, Cesar Picinini e, especialmente, à querida mestre de muitos entre nós, Ângela Biaggio, pioneira dos estudos em Psicologia do Desenvolvimento no país, que não está mais conosco, mas que nos deixou, assim como tantos outros mestres, uma herança e nos ensinou, assim como eles, que a herança pode ser transformada, levando-nos como discípulos a não ter medo de ousar e de inovar, legado que, também nós, devemos deixar aos nossos alunos e às novas gerações, no exercício do nosso gratificante ofício de formá-los bem para o ensino e para a pesquisa.

**Referências**

Bastos, A.V.; Tomanari, G. A. Y.; Trindade, Z.A. (2017). *Relatório de Avaliação Trienal – Psicologia.* Brasília: CAPES.

Gomes, W. B., & Hutz, C. (1987). A prática clínica e o trabalho acadêmico: Uma entrevista com a psicóloga Terezinha Féres Carneiro. *Psicologia: Reflexão e Crítica, 2*(1/2),73-78.

Féres-Carneiro, T. (2008). Pesquisa e prática clínica: construindo articulações teóricas. *Psicologia: Reflexão e Crítica*, 21(3), 349-358.

Féres-Carneiro, T.; Bastos, A.V.; Feitosa, M. A. G.; Seidl-de-Moura, M. L.; Yamamoto, O. W. (2010). Lacunas, metas e condições para aexpansão da pós-graduação em psicologia no país. *Psicologia: Reflexão e Crítica*, 23(1), 1-14.

Gomes, W. B., & Hutz, C. (2010). Anotações históricas e conceituais sobre o Programa de Pós-graduação em Psicologia da Universidade Federal do Rio Grande do Sul. *Psicologia: Reflexão e Crítica*, 23(1), 47-57.

Féres-Carneiro, T. (2017). 50 anos do Programa de Pós-graduação em Psicologia da PUC-Rio: uma história de pioneirismo, inovação e resiliência. *Psicologia Clínica*, (29(1), 1-6.

1. E-mail de contato: teferca@puc-rio.br [↑](#footnote-ref-1)
